# Willingness, perceived facilitators and barriers to use remote care among healthcare professionals – a cross-sectional study

**DOI:** 10.1186/s12913-023-10301-4

**Published:** 2023-11-27

**Authors:** Emil Eirik Kvernberg Thomassen, Inger Jorid Berg, Eirik Klami Kristianslund, Anne Therese Tveter, Nina Østerås

**Affiliations:** Centre for treatment of Rheumatic and Musculoskeletal Diseases (REMEDY), Diakonhjemmet Hospital, Oslo, Norway

**Keywords:** Remote care, Rheumatology, Inflammatory joint Disease, Healthcare professionals, Facilitators and barriers, Digital health

## Abstract

**Background:**

Remote care has the potential of improving access to timely care for people with inflammatory joint diseases (IJD), but there is limited knowledge on how this approach is regarded by healthcare professionals (HCP). This study aimed to examine willingness, perceived facilitators, and barriers to use remote care among HCP.

**Methods:**

Employees at 20 rheumatology departments in Norway received a digital survey containing 16 statements regarding willingness, perceived facilitators and barriers to use remote care. Statements were scored using numeric rating scales (NRS, 0–10, 10 = strongly agree), and analysed in linear regression models. Open-ended responses with participant-defined facilitators and barriers were analysed using qualitative manifest analysis.

**Results:**

A total of 130 participants from 17 departments completed the survey. The majority of participants were 45 years or older (*n* = 84, 54%), 54 (42%) were medical doctors, 48 (37%) nurses, and 27 (21%) were allied healthcare professionals, clinical leaders, or secretaries. A high willingness to use remote care was observed (median NRS: 9, IQR 8–10). The facilitator statement with the highest score was that patients save time and costs by using remote care, whereas the barrier statement with the highest score was the lack of physical examination. Willingness to use remote care was positively associated with the belief that patients wish to use it (β: 0.18, 95% CI: 0.00, 0.34), that patients in remission need less hospital visits (β: 0.30, 95% CI: 0.16, 0.43), and if remote care is widely adopted by co-workers (β: 0.27, 95% CI: 0.15, 0.39). Willingness was negatively associated with mistrust in the technical aspects of remote care (β: -0.26, 95% CI:-0.40, -0.11), and lack of physical examination (β: -0.24, 95% CI: -0.43, -0.06). The open-ended responses showed that technological equipment, eligible patients, user-friendly software, adequate training and work flow could be facilitators, but also that lack of these factors were considered barriers to use remote care.

**Conclusion:**

This study showed that HCP have a high willingness to use remote care, and provides important new knowledge on perceived facilitators and barriers among HCP relevant for implementation of remote care for eligible patients with IJD.

**Supplementary Information:**

The online version contains supplementary material available at 10.1186/s12913-023-10301-4.

## Introduction

Inflammatory joint diseases (IJDs) such as rheumatoid arthritis (RA) and spondyloarthritis (SpA), represent high-burden, chronic diseases requiring frequent follow-up from specialist healthcare in order to obtain and maintain low disease activity [1–3]. The shortage of healthcare professionals presents a challenge for providing timely management of IJDs for healthcare services, making efficient management of IJD patients crucial [4].

Implementation of remote care may alleviate the shortage of health professionals in IJD-management [5]. Remote care comprises technological modalities such as telephone and video consultations, synchronic/asynchronous chat, and monitoring of electronic patient reported outcomes (ePROs), as alternatives for out-patient visits [6]. Current research on remote care indicate an acceptance among patients as well as cost-effectiveness compared to standard care [7–9]. Despite possible advantages of remote care and positive experiences during the COVID-19 pandemic [10], there is a lack of knowledge regarding facilitators and barriers among health professionals on use of remote care for patients with IJD in a non-pandemic setting.

Remote care is in an early phase, and research on facilitators and barriers is required in order to integrate and adopt remote care into standard practice [11–14]. Earlier studies on facilitators and barriers to remote care in rheumatology have primarily focused on experiences from rheumatologists during the COVID-19 pandemic [10, 15, 16]. Since these, previous studies were conducted during the COVID-19 pandemic when physical consultations were limited to a minimum, there may be a bias towards more positive attitudes concerning remote care. As a multidisciplinary approach is recommended for the management of IJDs, and the different occupations play different roles, there is a need to investigate facilitators and barriers among a broader audience, including medical doctors, nurses, allied healthcare professionals (AHP), secretaries and clinical leaders in rheumatology departments [17, 18]. While it has previously been shown that higher age is associated with lower degrees of willingness to use remote care [19], this finding needs to be further investigated.

The primary aim of this study was to assess the degree of willingness, as well as perceived facilitators and barriers to use of remote care among medical doctors, nurses, AHPs, clinical leaders and secretaries at departments of rheumatology throughout Norway. The secondary aims were to explore associations between willingness to use remote care and perceived facilitators and barriers, including differences in perceived facilitators and barriers based on the participants’ occupations and age groups.

## Method and materials

### Study design

In this cross-sectional study, employees at departments of rheumatology throughout Norway were invited to complete an anonymous digital survey regarding perceived barriers and facilitators to use of remote care. The participants were recruited by convenience sampling using three different approaches: (1) Employees at one department of rheumatology (Diakonhjemmet Hospital) were invited to complete the questionnaire during a seminar in November 2021, (2) Invitations to complete the questionnaire were posted in two Facebook-groups, one for medical doctors and one for rheumatology nurses, and (3) Invitations were emailed to all 20 head of departments of rheumatology in Norway including a request to encourage the employees to complete the survey. The data collection was completed in April 2022. Ethical aspects and participant confidentiality were approved by the Data Protection Officer at Diakonhjemmet Hospital on October 13, 2021. The study ensured participant anonymity, making it exempt from the Norwegian Health Research Act and the further need of approval from the Regional Committee for Medical and Health Research Ethics.

The survey comprised a 22-item questionnaire developed by two of the authors (EEKT, NØ) and included statement-based items derived from previously identified facilitators and barriers to remote care [20–22]. Six items concerned characteristics of the participants; occupation (medical doctor vs nurse vs AHP/leader/secretary), age (< 45 vs ≥ 45 years), name of the department of rheumatology, and self-reported frequency of use of remote care modalities in clinical practice such as telephone, video consultation and ePROMS (not relevant/never/some times a year/monthly/weekly/daily). Due to participant anonymity, demographical characteristics were limited to age groups and occupation. The subsequent 14 items comprised six facilitators and eight barriers previously identified in the literature and were formulated as statements. The final two items consisted of statements regarding the participants’ willingness to use remote care: *“Our clinic should start using remote care”* and “*I think that remote care will be a part of healthcare services in the future*”. The participants rated level of agreement with the 16 statements using a 11-point numeric rating scales (NRS) from 0=”Strongly disagree” to 10=“Strongly agree”, with 5=”Neutral”. Two open-ended questions were included at the end of the questionnaire to capture participant-defined facilitators and barriers. Here, the participants were encouraged to write keywords on factors they believed would increase or decrease the probability of using remote care in clinical practice.

### Data collection

Survey data was collected online through Nettskjema (nettskjema.no), which is delivered and hosted by the University of Oslo. In the introductory part of the questionnaire, the participants were given a brief description of the aim of the study and examples illustrating ways of delivering remote care, e.g., monitoring ePROs, video consultations. A definition of remote care was also provided. In addition, participants were instructed to envision eligible patients for remote care: patients with low disease activity, stable treatment and that are considered eligible for remote care (e.g., absence of cognitive impairments, speech-impediments, or multi-comorbidity).

### Statistical analyses

Demographic data are presented with frequencies and percentages. The Chi-square test was applied to assess the association between age- and occupation groups. The median NRS scores and interquartile range (IQR) for the 14 statements on perceived facilitators and barriers are visualised in a boxplot, showing median score in descending order. The two statements on the degree of willingness to use remote care were divided by occupation in a separate boxplot. Due to a low number of participants in the AHP/leader/secretary group, the subgroup analyses only included medical doctors and nurses. Analyses of between-group differences in median NRS scores on the 14 statements for the medical doctors and nurses, and the two age groups were performed using the Wilcoxon rank sum-test. Multivariable linear regression analyses were used to assess associations between the degree of willingness to use remote care by the statement: *“Our clinic should start using remote care”* (dependent variable) and the six facilitators and eight barriers in two separate models, adjusted for age and occupation. The residuals of the models were inspected for distribution and multicollinearity. The significance level was set to < 0.05, and the analyses were performed using STATA 16.4.

Open-ended responses were analysed using a qualitative manifest analysis [23] as used by Wode, Henriksson [24]. Six of the open-ended responses were ambiguous and were therefore excluded from the analysis. An inductive analysis with categorisation was completed by two of the authors (EEKT, NØ), and then discussed with the rest of the co-authors leading to a reduction of categories. The frequency of keywords used by participants were summarised for each category, and selected responses were translated from Norwegian to English to provide examples.

## Results

**Fig. 1:**
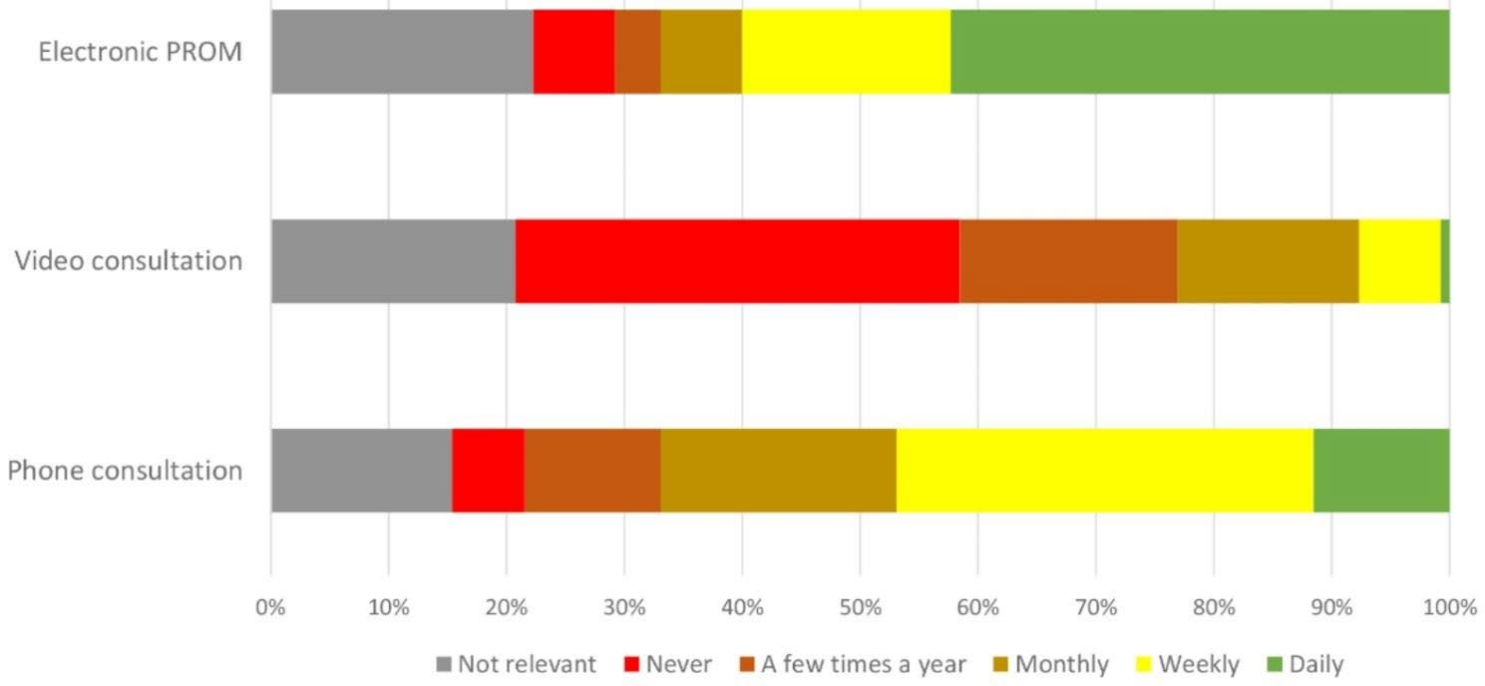
Healthcare professionals’ self-reported use of different remote care modalities (*n* = 130)

**Fig. 2:**
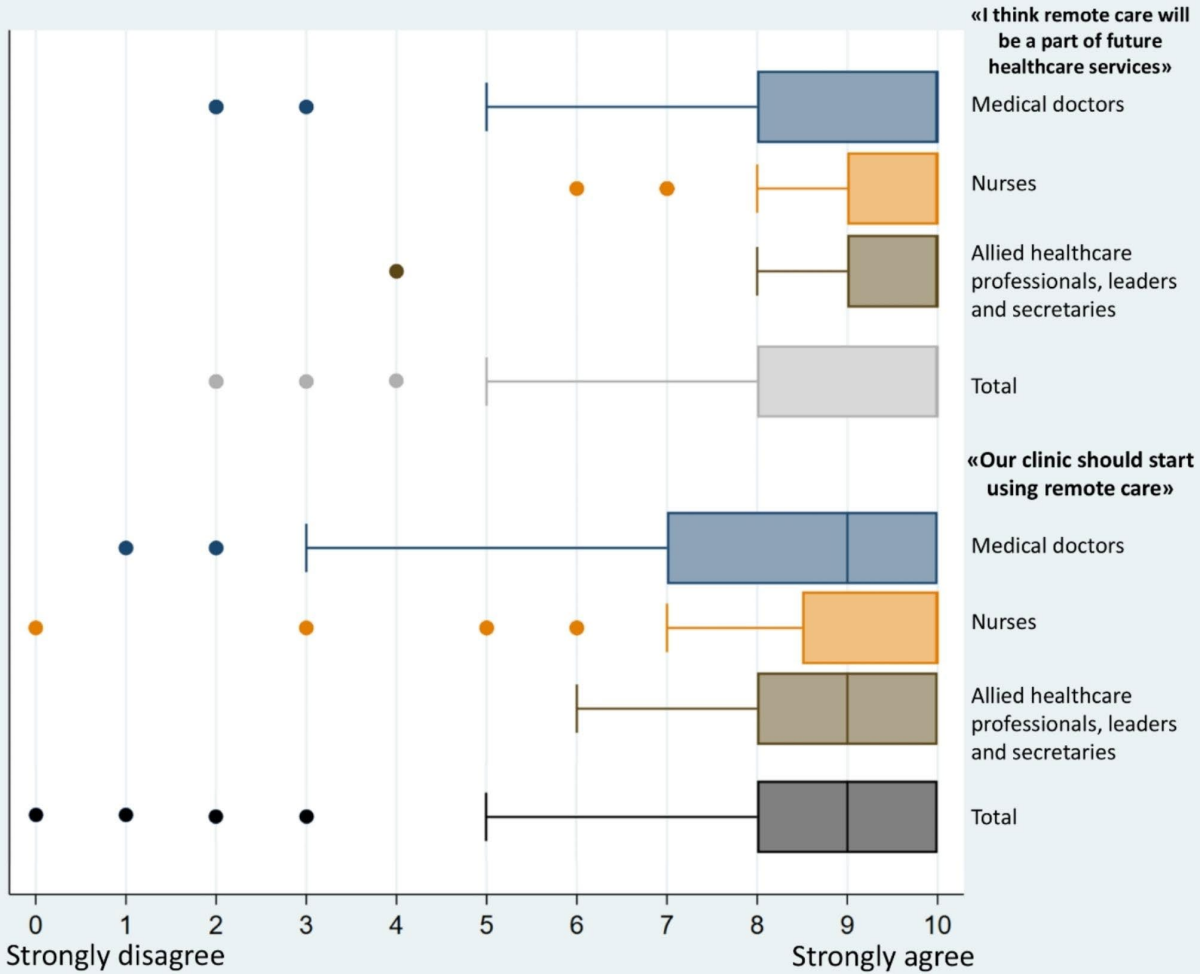
Healthcare professionals’ attitudes towards remote care by occupation group (*n* = 129)

More than 550 employees from all 20 departments of rheumatology in Norway were invited to participate, with 130 participants (~ 24%) from 17 departments completing the questionnaire (Table 1). A larger proportion of the participants were from one single department (55%). There was no statistically significant difference in age across the occupation groups (X^2^: 1.4, *p* = 0.5). Nearly half of the participants reported that they conducted weekly or daily phone consultations, while weekly or daily use of video consultation was reported by 8% (Fig. 1). ePROMs were used on a daily or weekly basis by 60% of the participants. While a few participants reported a low degree of willingness to use remote care, the vast majority of participants reported a high degree of willingness to use remote care and belief that remote care will be part of future healthcare with minor differences in median scores between the three occupation groups (Fig. 2).

**Table 1:** Characteristics (age and profession) of the participants (*n* = 130)

	Total	Age groups	
Occupation*		< 45 years	≥ 45 years
Medical doctor (including rheumatologist), *n* (%)	54 (42%)	17 (31%)	37 (69%)
Nurse, *n* (%)	48 (37%)	16 (33%)	32 (67%)
Allied healthcare, clinical leader or secretary, *n* (%)	27 (21%)	12 (44%)	15 (56%)

*=129 due to one missing response regarding occupation

**Fig. 3:**
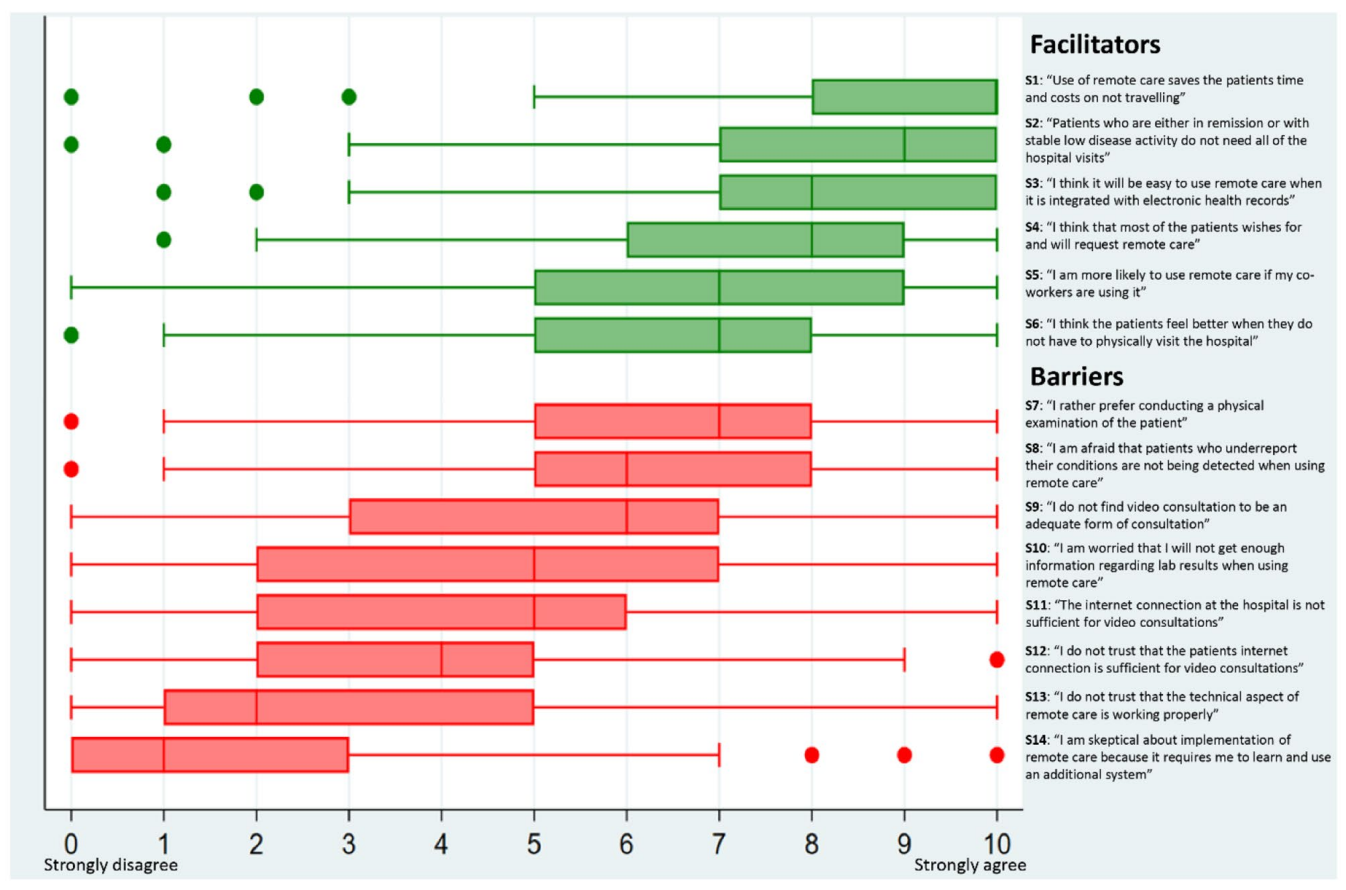
Healthcare professionals’ agreement on the statement-based perceived facilitators and barriers (*n* = 130)

The median scores on the perceived facilitators were in general higher than the median scores on barriers (Fig. 3). The facilitator regarding that the patients would save time and costs if they did not need to travel for hospital visits had the highest median score (median 10), while the highest median score for barriers was observed for the statement “I rather prefer conducting a physical examination of patient (median 7). In adjusted analyses a higher degree of willingness to use remote care was positively associated with the following statements: “I think that patients want to use remote care”, “Patients who are either in remission or with stable low disease activity do not need all of the hospital visits”, and “I am more likely to use remote care if my co-workers are using it” (Table 2). Being 45 years or older was also significantly associated with a higher degree of willingness to use remote care (Table 2). The second adjusted regression model showed that the barriers “I do not trust that the technical aspects of remote care is working properly” and “I rather prefer conducting a physical examination of the patient” were significantly negatively associated with a lower degree of willingness to use remote care (Table 3).

**Table 2:** The association between healthcare professionals’ willingness to use remote care and statement-based facilitators

Dependent variable Statement: “Our clinic should start using remote care”	Unadjusted model			Adjusted model		
	β-coefficient	95% CI	*p*-value	β-coefficient	95% CI	*p*-value
**Facilitators:**						
Use of remote care saves the patients time and costs on not travelling	0.38	0.18, 0.58	0.000	-0.10	-0.29, 0.08	0.271
I think that most of the patients wish for and will request remote care	0.45	0.30, 0.60	0.000	0.18	0.02, 0.34	0.023
I think it will be easy to use remote care when it is integrated with electronic health records	0.48	0.32, 0.63	0.000	0.07	-0.09, 0.25	0.393
Patients who are either in remission or with stable low disease activity do not need all of the hospital visits	0.45	0.32, 0.58	0.000	0.30	0.16, 0.43	< 0.01
I think the patients feel better when they do not have to physically visit the hospital	0.39	0.24, 0.54	0.000	0.08	-0.06, 0.23	0.254
I am more likely to use remote care if my co-workers are using it	0.43	0.30, 0.56	0.000	0.27	0.15, 0.39	< 0.01
Age						
< 45 years	ref.					
45 years or more	0.547	-0.20, 1.29	0.100	0.78	0.21, 1.35	< 0.01
Occupation						
Medical doctor	ref.					
Nurse	0.678	-0.132, 1.488	0.100	0.12	-0.48, 0.74	0.686
AHP/leader/secretary	0.574	-0.388, 1.537	0.240	0.64	-0.07, 1.36	0.077

Univariate and multivariate linear regression analyses

**Table 3:** The association between healthcare professionals’ willingness to use remote care and statement-based barriers

Dependent variable Statement: “Our clinic should start using remote care”	Unadjusted model			Adjusted model		
	β-coefficient	95% CI	*p*-value	β-coefficient	95% CI	*p*-value
**Barriers:**						
I am skeptical of the introduction of remote care since it requires that I have to learn and use an additional system	-0.20	-0.35, -0.04	0.012	-0.10	-0.25, 0.04	0.172
I do not trust that the technical aspect of remote care is working properly	-0.31	-0.44, -0.18	0.000	-0.26	-0.40, -0.11	< 0.01
The internet connection at the hospital is not sufficient for video consultations	0.00	-0.12, 0.12	0.99	0.12	-0.00, 0.25	0.051
I do not trust that the patient’s internet connection is sufficient for video consultations	-0.17	-0.32, -0.03	0.014	-0.02	-0.18, 0.12	0.708
I do not find video consultation to be an adequate form of consultation	-0.27	-0.39, -0.15	0.000	-0.13	-0.28, 0.02	0.108
I am afraid that patients who underreport their conditions are not being detected when using remote care	-0.19	-0.33, -0.05	0.006	0.03	-0.11, 0.19	0.632
I rather prefer conducting a physical examination of the patient	-0.37	-0.51, -0.23	0.000	-0.24	-0.43, -0.06	< 0.01
I am worried that I will not get enough information regarding lab results when using remote care	-0.72	-0.208, 0.063	0.293	0.02	-0.10, 0.15	0.734
Age						
< 45 years	ref.					
45 years or more	0.547	-0.20, 1.29	0.100	0.42	-0.28, 1.13	0.239
Occupation						
Medical doctor	ref.					
Nurse	0.678	-0.132, 1.488	0.100	-0.44	-1.23, 0.35	0.275
AHP/leader/secretary	0.574	-0.388, 1.537	0.240	0.28	-0.61, 1.17	0.533

Univariate and multivariate linear regression analysis

**Fig. 4:**
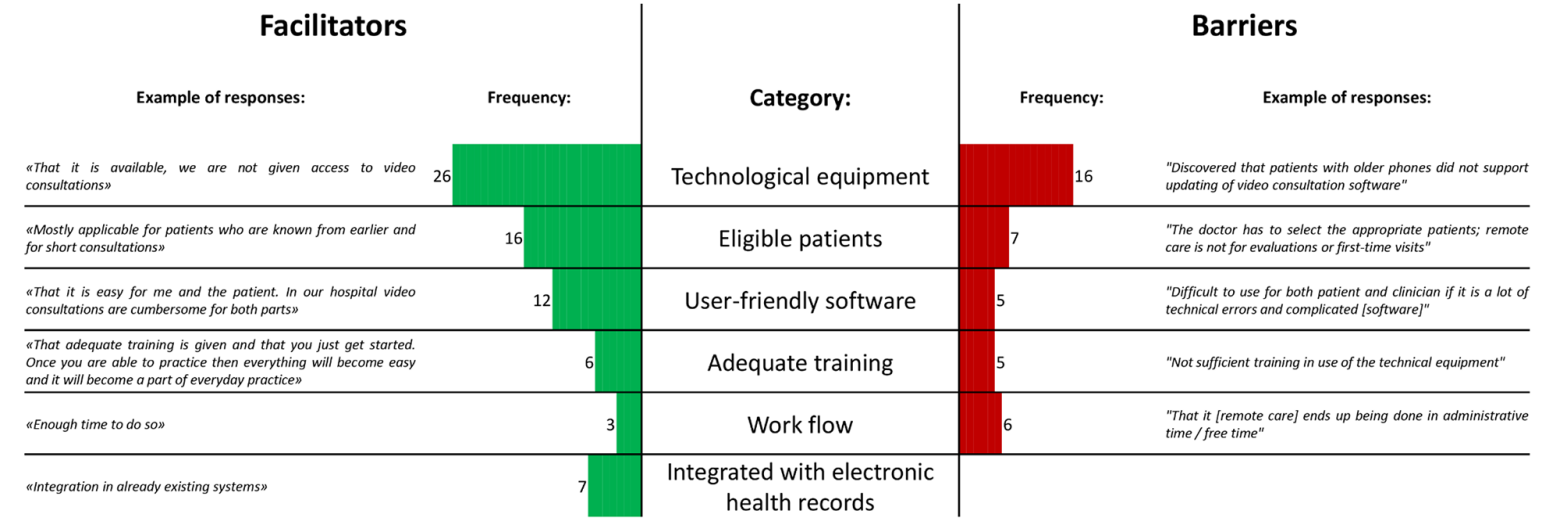
Open-ended facilitators and barriers sorted by categories with frequencies and examples of responses

The open-ended responses on barriers and facilitators were completed by 67 (51%) participants reporting a total of 67 facilitators and 47 barriers. The most frequently mentioned categories, serving as both facilitators and barriers to use of remote care, were technological equipment, eligible patients, user-friendly software, adequate training, and workflow. Integration of the remote care software with electronic health records was reported as a facilitator (Fig. 4).

There were few statistically significant differences in subgroup analysis between occupations and age groups as shown in Additional files 1 and 2.

## Discussion

In the current study, healthcare professionals, clinical leaders, and secretaries in rheumatology care reported a high degree of willingness to use remote care. The most important perceived facilitators for use of remote care were the belief that patients in remission or with low disease activity may need less hospital visits, that the patients would prefer remote care, and if remote care is widely adopted by co-workers. Among perceived barriers, the most important were a mistrust in the technological aspects of remote care and that healthcare professionals preferred conducting a physical examination of the patients. Open-ended facilitators and barriers included technological equipment, eligible patients, user-friendly software, adequate training, and workflow, acting as both facilitators and as barriers to remote care, whereas integration of remote care software in electronic health records was reported to be a facilitator.

In line with other studies, the participants in this study reported infrequent use of video consultations, while phone consultations and monitoring of ePROMS were more commonly used [10, 25]. The explanation for the high proportions of participants using ePROMS on a weekly or daily basis in this study is likely related to the use of a software made for collecting IJD-relevant ePROMS in relation to a consultation. This software is implemented in all Norwegian departments of rheumatology. In the open-ended responses, many participants reported both lack and poor quality of technological equipment, which may contribute to limited use of video consultations. The use of phone consultations was also shown to be acceptable as an alternative to face-to-face visits during the COVID-19 pandemic, and favourable over video by patients, which might explain the frequent use of phone consultations [25, 26]. Administrating video consultations are in general considered more time-consuming compared to phone consultations, and technical errors are more likely to occur [27], which might provide some explanation to the current study’s infrequent use of video consultations.

Regardless of the infrequent use of video consultations, participants reported a high degree of willingness to use remote care. Similar levels of willingness have been reported in some studies [10, 26, 28], with contrasting findings in studies reporting that remote care and video consultations were considered to be inferior to regular face-to-face consultations [29, 30]. While the previous studies reporting positive attitudes were conducted during the COVID-19 pandemic, the current survey was completed later during the pandemic when face-to-face visits at outpatient clinics were standard follow-up.

Higher degree of willingness to use remote care was significantly associated with the statement “Patients who are either in remission or with stable low disease activity do not in need all of the hospital visits”. This may suggest which patient group the participants considered to be eligible for receiving remote care. In accordance with this, a recent study found that 34% of outpatient visits by patients with axial spondyloarthritis were considered unnecessary by rheumatologist, and the authors suggested that triaging patients can be completed remotely [31]. This finding is also in compliance with EULAR’s points-to-consider for remote care, which states that patients with low disease activity could receive remote care as an alternative to face-to-face visits, but that newly diagnosed patients should have at least one physical consultations before being offered remote care [11]. This has also has been proposed in the wider discussion regarding remote care in rheumatology [6, 14, 32]. The inability to perform a physical examination was identified as a barrier in our study. This has been confirmed by another study [10], and poor safety may be of concern in terms of monitoring disease activity when managing IJDs. However, one study on patients with systemic lupus erythematosus found no difference in disease activity between patients receiving care remotely or in-hospital [33]. Other studies have shown equal safety in terms of adverse events, but that the aspect of safety needs further research in order to be established [34]. Despite evidence on safety, the results from this study shows that lack of physical examination is a barrier for using remote care for patients with IJD and that adaptations to accommodate the lack of physical examination are necessary.

A widespread adoption of remote care among co-workers was deemed important by the participants. An earlier review showed that endorsement by senior co-workers facilitated the use of technology-based interventions among healthcare professionals [35]. There is also evidence suggesting that change facilitated by local opinion leaders may serve as an effective implementation strategy [36]. Receiving feedback and discussing clinical issues between co-workers may also lead to changes in healthcare professional’s uptake of clinical practice guidelines [37].

Effective technological solutions were important for the degree of willingness to use remote care. Defective technology has proven to be a major factor for mistrust in remote care [38]. However, as the advancement in technology is moving rapidly the difficulties of implementing remote care may be more an issue of regulatory and government issue rather than the current technology [14]. When implementing remote care in clinical practice, all stakeholders including developers should therefore be included in the process [11, 38]. This may counteract some of the healthcare professionals’ mistrust by allowing for adapted software and technology and further increased use of remote care.

### Strengths and limitations

Strengths in this study include that participants represented most of the departments invited to the survey, providing diversity between rural and urban hospitals, which may strengthen the generalisability of the results. The relative high number of participants also allowed for multivariate regression models with reduced risk of overfitting. By using a combination of predefined statements and participants-defined open-ended facilitators and barriers, we were able to elaborate from the perceived facilitators and barriers previously defined in the literature.

Surveys are prone to selection bias, and this assumption may be strengthened by the high level of willingness in our results – which might imply that mostly healthcare professionals with high interest in remote care participated. The use of a self-made questionnaire is a limitation in the current study. However, statements that constituted facilitators and barriers were identified from systematic reviews and participants were given the possibility to add facilitators and barriers in the open-ended items. In order to secure participant anonymity, the collection of demographic data in this study was very limited. More demographic data would have given the opportunity to describe the study sample and allowed for additional sub-group analyses. As the participants were instructed to envision eligible patients with IJDs in the introductory part of the survey, caution should be applied in generalising the results to other diagnosis. The relatively low response rate in this study introduces a potential selection bias and reduces the external validity of the results. Additionally, a larger proportion of participants belonged to a single department, which may reduce the nationwide generalisability of the results in the current study.

### Implications

This study demonstrated a high degree of willingness to use remote care for patients with IJDs among healthcare professionals, clinical leaders and secretaries. The perceived facilitators and barriers highlighted in this study should be considered when implementing remote care in clinical practice. Although the survey instructions directed the participants to consider patients with rheumatic disease, the findings may be applicable for the implementation of remote care beyond rheumatology due to the study’s focus on patients with well-treated chronic conditions.

## Conclusion

This study indicated a high degree of willingness among healthcare professionals to integrate remote care in clinical practice for eligible patients with IJDs. For a successful implementation, the implementation strategy should be tailored to the healthcare professionals’ perceived facilitators and barriers to remote care as identified by our study. For future research, conducting studies with a qualitive approach would provide further in-depth knowledge to inform implementation strategies, including patients’ perspectives on remote care.

### Electronic supplementary material

Below is the link to the electronic supplementary material.

Supplementary Material 1: Differences in scores between medical doctors (*n* = 54) and nurses (*n* = 48) facilitators and barriers

Supplementary Material 2: Differences in score between age groups on facilitators and barriers

## Data Availability

The dataset generated and analysed during the current study is available from the corresponding author on reasonable request.

## Abbreviations

AHP: Allied healthcare professionals
COVID-19: Corona virus disease
ePROS: electronic patient reported outcomes
EULAR: European Alliance of Associations for Rheumatology
IJD: Inflammatory joint diseases
IQR: Interquartile range
NRS: Numeric rating scales
RA: Rheumatoid arthritis
SpA: Spondyloarthritis
