# Supplementary material for: Willingness, perceived facilitators and barriers to use remote care among healthcare professionals – a cross-sectional study
Source: BMC Health Serv Res. 2023 Nov 27;23:1307. doi: 10.1186/s12913-023-10301-4 (PMC10683299; doi:10.1186/s12913-023-10301-4)
Supplement: Supplementary file 1 — Supplementary Material 1: Differences in scores between medical doctors (n = 54) and nurses (n = 48) facilitators and barriers [file 12913_2023_10301_MOESM1_ESM.docx]

**Supplementary material 1**. Differences in scores between medical doctors (n= 54) and nurses (n= 48) facilitators and barriers

| **Statement** | **Medical doctors**  (median (IQR)) | **Nurse**  (median (IQR)) | Wilcoxon rank sum test |
| --- | --- | --- | --- |
| **S1**: «Use of remote care saves the patients time and resources on not travelling» | **9 (8-10)** | **10 (9.5-10)** | **Z =-3.249, p=<0.01** |
| **S2**: « Patients who are either in remission or with stable low disease activity do not need all of the hospital visits » | 8.5 (6-10) | 9 (8-10) | Z=-1.567, p=0.117 |
| **S3**: «I think it will be easy to use remote care when it is integrated with electronic health records» | **8 (5-10)** | **9 (8-10)** | **Z=-2.160, p=0.030** |
| **S4**: «I think that most of the patients wishes for and will request remote care» | 7 (6-9) | 8 (6-10) | Z=-1.364, p=0.172 |
| **S5**: «I am more likely to use remote care if my colleagues are using it» | 7 (5-9) | 7 (5-10) | Z=0.970, p=0.332 |
| **S6**: «I think the patients feel better when they do not have to physically visit the hospital» | 6 (5-8) | 7 (6-9.5) | Z=-2.000, p=0.456 |
| **S7**: «I rather prefer conducting a physical examination of the patient» | **7 (5-8)** | **5 (4-7)** | **Z=2.944, p=<0.01** |
| **S8**: « I am afraid that patients who underreport their conditions are not being detected when using remote care» | **7 (6-9)** | **7 (4-8)** | **Z=2.190, p=0.028** |
| **S9:** «I do not find video consultation to be an adequate form of consultation» | **7 (5-9)** | **3 (2-6.5)** | **Z=4.553, p=<0.001** |
| **S10**: «I am worried that I will not get enough information regarding lab results when using remote care» | **5 (2-7)** | **3 (1-6)** | **Z=2.049, p=0.040** |
| **S11**: «The internet connection at the hospital is not sufficient for video consultations» | 5 (1-6) | 5 (2-5) | Z=0.371, p=0.711 |
| **S12**: « I do not trust that the patient’s internet connection is sufficient for video consultations» | **4 (2-6)** | **2 (1-5)** | **Z=2.276, p=0.022** |
| **S13**: «I do not trust that the technical aspect of remote care is working properly» | **2.5 (1-5)** | **1 (0-2)** | **Z=3.236, p=<0.01** |
| **S14**: «I am skeptical about implementation of remote care because it requires me to learn and use an additional system» | **2 (0-4)** | **0 (0-2)** | **Z=2.866, p=<0.01** |

Wilcoxon rank sum test
