# Supplementary material for: Willingness, perceived facilitators and barriers to use remote care among healthcare professionals – a cross-sectional study
Source: BMC Health Serv Res. 2023 Nov 27;23:1307. doi: 10.1186/s12913-023-10301-4 (PMC10683299; doi:10.1186/s12913-023-10301-4)
Supplement: Supplementary file 2 — Supplementary Material 2: Differences in score between age groups on facilitators and barriers [file 12913_2023_10301_MOESM2_ESM.docx]

| Statement | **Age <45** (median (IQR)) | Age >45 (median (IQR)) | Wilcoxon rank sum test |
| --- | --- | --- | --- |
| **S1**: «Use of remote care saves the patients time and resources on not travelling» | 10 (8-10) | 10 (9-10) | z= -1.003, p=0.315 |
| **S2**: « Patients who are either in remission or with stable low disease activity do not need all of the hospital visits» | 9 (7-10) | 8 (7-10) | z=0.547, p=0.584 |
| **S3**: «I think it will be easy to use remote care when it is integrated with electronic health records» | 8 (8-9) | 8 (6-10) | z=0.137, p=0.890 |
| **S4**: «I think that most of the patients wishes for and will request remote care» | 7 (6-8) | 8 (6-10) | z=-1.814, p=0.069 |
| **S5**: «I am more likely to use remote care if my colleagues are using it» | 8 (5-9) | 7 (5-10) | z=0.896, p=0.370 |
| **S6**: «I think the patients feel better when they do not have to physically visit the hospital» | 7 (5-8) | 7 (5-8) | z=1.038, p=0.299 |
| **S7**: « I rather prefer conducting a physical examination of the patient» | 6 (5-8) | 5 (5-7) | z=1.055, p=0.291 |
| **S8**: « I am afraid that patients who underreport their conditions are not being detected when using remote care» | 7 (5-8) | 7 (5-8) | z=0.305, p=0.760 |
| **S9:** «I do not find video consultation to be an adequate form of consultation» | 6 (4-7) | 6 (3-8) | z=-0.538, p=0.590 |
| **S10**: «I am worried that I will not get enough information regarding lab results when using remote care» | 5 (3-6) | 5 (2-7) | z=-0.101, p=0.919 |
| **S11**: «The internet connection at the hospital is not sufficient for video consultations » | **5 (5-7)** | **5 (1-5)** | z=3.020, **p=0.002** |
| **S12**: « I do not trust that the patient’s internet connection is sufficient for video consultations» | **5 (3-7)** | **3 (2-5)** | z=2.410, **p=0.016** |
| **S13**: «I do not trust that the technical aspect of remote care is working properly» | **3 (1-6)** | **2 (0-4)** | z=2.700, **p=0.006** |
| **S14**: «I am skeptical about implementation of remote care because it requires me to learn and use an additional system» | 1 (0-2) | 1 (0-3) | z=-0.248, p=0.804 |

**Supplementary material 2**. Differences in score between age groups on facilitators and barriers

Wilcoxon rank sum test
